# Ethyl­enediammonium dichloro­iodide chloride

**DOI:** 10.1107/S1600536809039038

**Published:** 2009-10-03

**Authors:** Li-Zhuang Chen

**Affiliations:** a School of Material Science and Engineering, Jiangsu University of Science and Technology, Zhenjiang 212003, People’s Republic of China

## Abstract

The asymmetric unit of the crystal structure of the title compound, C_2_H_10_N_2_^2+^·Cl_2_I^−^·Cl^−^, contains two ethyl­ene­diammonium cations, two [ICl_2_]^−^ anions and two Cl^−^ anions, of which one cation, one [ICl_2_]^−^ anion and one Cl^−^ anion have site symmetry 2, with the mid-point of the C—C bond of the cation, the I atom of [ICl_2_]^−^ anion and the Cl^−^ anion located on the twofold rotation axes. The two independent cations show different conformations, the N—C—C—N torsion angles being 160.1 (2) and −73.1 (4)°. The crystal structure is stabilized by extensive inter­molecular N—H⋯Cl hydrogen bonding.

## Related literature

For general background to combining protonated aromatic nitro­gen bases with halide or polyhalide ions, see: Tucker & Kroon (1973 ▶); Bandoli *et al.* (1978 ▶). For Cl—I bond lengths and Cl–I–Cl bond angles, see: Lang *et al.* (2000 ▶); Wang *et al.* (1999*a* ▶,*b* ▶).
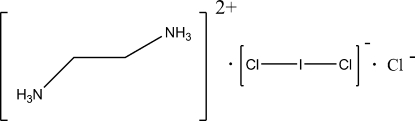


## Experimental

### 

#### Crystal data

C_2_H_10_N_2_^2+^·Cl_2_I^−^·Cl^−^
*M* *_r_* = 295.37
Monoclinic, 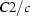

*a* = 8.565 (2) Å
*b* = 16.2186 (15) Å
*c* = 19.9631 (16) Å
β = 101.164 (16)°
*V* = 2720.8 (7) Å^3^
*Z* = 12
Mo *K*α radiation
μ = 4.34 mm^−1^
*T* = 293 K
0.36 × 0.30 × 0.28 mm

#### Data collection

Rigaku SCXmini diffractometer
Absorption correction: multi-scan (*CrystalClear*; Rigaku, 2005 ▶) *T*_min_ = 0.230, *T*_max_ = 0.301
13418 measured reflections
3106 independent reflections
2821 reflections with *I* > 2σ(*I*)
*R*_int_ = 0.034

#### Refinement

*R*[*F* ^2^ > 2σ(*F* ^2^)] = 0.023
*wR*(*F* ^2^) = 0.056
*S* = 1.10
3106 reflections
114 parameters
H-atom parameters constrained
Δρ_max_ = 0.92 e Å^−3^
Δρ_min_ = −0.65 e Å^−3^

### 

Data collection: *CrystalClear* (Rigaku, 2005 ▶); cell refinement: *CrystalClear*; data reduction: *CrystalClear*; program(s) used to solve structure: *SHELXS97* (Sheldrick, 2008 ▶); program(s) used to refine structure: *SHELXL97* (Sheldrick, 2008 ▶); molecular graphics: *ORTEP-3 for Windows* (Farrugia, 1997 ▶); software used to prepare material for publication: *WinGX* (Farrugia, 1999 ▶).

## Supplementary Material

Crystal structure: contains datablocks I, global. DOI: 10.1107/S1600536809039038/xu2588sup1.cif

Structure factors: contains datablocks I. DOI: 10.1107/S1600536809039038/xu2588Isup2.hkl

Additional supplementary materials:  crystallographic information; 3D view; checkCIF report

## Figures and Tables

**Table 1:** Hydrogen-bond geometry (Å, °)

*D*—H⋯*A*	*D*—H	H⋯*A*	*D*⋯*A*	*D*—H⋯*A*
N1—H1*A*⋯Cl1^i^	0.89	2.65	3.410 (3)	144
N1—H1*A*⋯Cl3^i^	0.89	2.76	3.341 (3)	124
N1—H1*B*⋯Cl4	0.89	2.27	3.136 (2)	164
N1—H1*C*⋯Cl5^i^	0.89	2.27	3.148 (3)	168
N2—H2*A*⋯Cl4^ii^	0.89	2.38	3.232 (3)	161
N2—H2*B*⋯Cl5^iii^	0.89	2.26	3.123 (3)	162
N2—H2*C*⋯Cl3^ii^	0.89	2.40	3.246 (3)	159
N3—H3*A*⋯Cl3^iv^	0.89	2.42	3.297 (2)	167
N3—H3*B*⋯Cl5	0.89	2.32	3.144 (3)	154
N3—H3*C*⋯Cl1^ii^	0.89	2.49	3.319 (2)	155

Symmetry codes: (i) 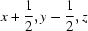; (ii) 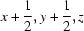; (iii) 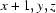; (iv) 
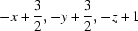
.

**Fig. 1.**
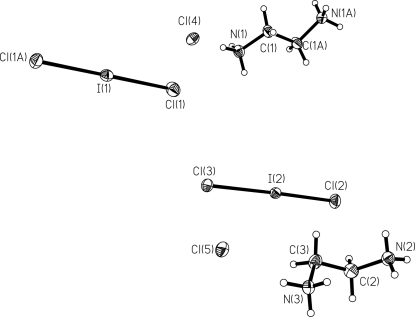
The structure of the title compound with atom labels. Displacement ellipsoids were drawn at the 40% probability level [symmetry code: (i) -x, y, -z+1/2; (ii) 2-x, y, -z+1/2].

## supplementary crystallographic information

### Comment

Recently much attention has been devoted to combining protonated aromatic nitrogen bases with halide or polyhalide ions due to their interesting structural features (Tucker & Kroon, 1973; Bandoli *et al.*, 1978). In our laboratory, a compound containing diprotonated ethylenediamine and ICl_2_ anions has been synthesized, its crystal structure is reported herein.

The asymmetric unit of the title compound, [C_2_H_10_N_2_]^2+^.[ICl_2_]^-^.Cl^-^, (Fig. 1) consists of two diprotonated ethylenediammonium cations, two [ICl_2_]^-^ anions and two Cl^-^ anions. The dichloroiodide anion Cl1–I1–Cl1A has site symmetry 2 and is linear with Cl1—I1—Cl1A bond angle of 179.55 (4). The Cl1—I1 bond length is similar to the values of 2.5417 (11) to 2.5575 (10) Å reported by (Wang *et al.*, 1999*a*,*b*). In Cl2—I2—Cl3 anion, the I2—Cl3 bond length of 2.6790 (9) Å is longer than I2—Cl2 bond length of 2.4518 (10) Å. The Cl2—I2—Cl3 is also nearly linear, the Cl2—I2—Cl3 bond angle being 178.30 (3)°. The nearly linear Cl—I—Cl bonds are similar to those reported by Lang *et al.* (2000) and Wang *et al.* (1999*a*,*b*). The two independent cations show the different conformations, the N-C-C-N torsion angles being 160.1 (2) and -73.1 (4)°. The crystal structure is stabilized by intermolecular N—H···Cl hydrogen bonds (Fig. 2).

### Experimental

KI (0.33 g) and I_2_ (0.5 g) were dissolved in a mixed solution of ethanol (30 ml) and concentrated hydrochloric acid (10 ml, 36%). On addition of ethylenediamine (0.60 g) to the above solution, the mixture was stirred for 2 h, then filtered. The filtrate was left at room temperature to allow the solvent to evaporate. Yellow transparent block crystals were obtained after two weeks.

### Refinement

H atoms were placed in calculated positions with C—H = 0.97 Å and N—H = 0.89 Å, and refined using a riding model, with U_iso_(H) = 1.2U_eq_(C) and 1.5U_eq_(N).

### Crystal data

**Table:**

C_2_H_10_N_2_^2+^·Cl_2_I^−^·Cl^−^	*F*(000) = 1680
*M**_r_* = 295.37	*D*_x_ = 2.163 Mg m^−^^3^
Monoclinic, *C*2/*c*	Mo *K*α radiation, λ = 0.71073 Å
Hall symbol: -C 2yc	Cell parameters from 2821 reflections
*a* = 8.565 (2) Å	θ = 2.5–27.5°
*b* = 16.2186 (15) Å	µ = 4.34 mm^−^^1^
*c* = 19.9631 (16) Å	*T* = 293 K
β = 101.164 (16)°	Block, yellow
*V* = 2720.8 (7) Å^3^	0.36 × 0.30 × 0.28 mm
*Z* = 12	

### Data collection

**Table:**

Rigaku SCXmini diffractometer	3106 independent reflections
Radiation source: fine-focus sealed tube	2821 reflections with *I* > 2σ(*I*)
graphite	*R*_int_ = 0.034
Detector resolution: 13.6612 pixels mm^-1^	θ_max_ = 27.5°, θ_min_ = 2.5°
ω scans	*h* = −11→11
Absorption correction: multi-scan (*CrystalClear*; Rigaku, 2005)	*k* = −20→20
*T*_min_ = 0.230, *T*_max_ = 0.301	*l* = −25→25
13418 measured reflections	

### Refinement

**Table:**

Refinement on *F*^2^	Secondary atom site location: difference Fourier map
Least-squares matrix: full	Hydrogen site location: inferred from neighbouring sites
*R*[*F*^2^ > 2σ(*F*^2^)] = 0.023	H-atom parameters constrained
*wR*(*F*^2^) = 0.056	*w* = 1/[σ^2^(*F*_o_^2^) + (0.0271*P*)^2^] where *P* = (*F*_o_^2^ + 2*F*_c_^2^)/3
*S* = 1.10	(Δ/σ)_max_ < 0.001
3106 reflections	Δρ_max_ = 0.92 e Å^−^^3^
114 parameters	Δρ_min_ = −0.65 e Å^−^^3^
0 restraints	Extinction correction: *SHELXL97* (Sheldrick, 2008), Fc^*^=kFc[1+0.001xFc^2^λ^3^/sin(2θ)]^-1/4^
Primary atom site location: structure-invariant direct methods	Extinction coefficient: 0.00017 (4)

### Special details

**Table:**

Geometry. All e.s.d.'s (except the e.s.d. in the dihedral angle between two l.s. planes) are estimated using the full covariance matrix. The cell e.s.d.'s are taken into account individually in the estimation of e.s.d.'s in distances, angles and torsion angles; correlations between e.s.d.'s in cell parameters are only used when they are defined by crystal symmetry. An approximate (isotropic) treatment of cell e.s.d.'s is used for estimating e.s.d.'s involving l.s. planes.
Refinement. Refinement of *F*^2^ against ALL reflections. The weighted *R*-factor *wR* and goodness of fit *S* are based on *F*^2^, conventional *R*-factors *R* are based on *F*, with *F* set to zero for negative *F*^2^. The threshold expression of *F*^2^ > σ(*F*^2^) is used only for calculating *R*-factors(gt) *etc*. and is not relevant to the choice of reflections for refinement. *R*-factors based on *F*^2^ are statistically about twice as large as those based on *F*, and *R*- factors based on ALL data will be even larger.

### Fractional atomic coordinates and isotropic or equivalent isotropic displacement parameters (Å^2^)

**Table:**

	*x*	*y*	*z*	*U*_iso_*/*U*_eq_	
C1	0.9115 (3)	0.1716 (2)	0.24029 (15)	0.0472 (8)	
H1D	0.8758	0.2243	0.2199	0.057*	
H1E	0.8778	0.1289	0.2065	0.057*	
C2	1.0658 (4)	0.8169 (2)	0.43343 (16)	0.0412 (7)	
H2D	1.0988	0.8492	0.4747	0.049*	
H2E	1.1129	0.7626	0.4416	0.049*	
C3	0.8881 (4)	0.80844 (18)	0.41979 (16)	0.0415 (7)	
H3D	0.8539	0.7849	0.3747	0.050*	
H3E	0.8592	0.7700	0.4526	0.050*	
Cl1	0.30378 (10)	0.45106 (5)	0.26597 (4)	0.04654 (19)	
Cl2	1.16812 (9)	0.58195 (6)	0.42064 (4)	0.04530 (19)	
Cl3	0.57256 (8)	0.54598 (5)	0.41952 (3)	0.03576 (16)	
Cl4	0.5000	0.23483 (7)	0.2500	0.0401 (2)	
Cl5	0.47720 (9)	0.79063 (5)	0.40942 (4)	0.04283 (18)	
I1	0.0000	0.450435 (17)	0.2500	0.03375 (8)	
I2	0.88263 (2)	0.564990 (11)	0.418170 (9)	0.02956 (7)	
N1	0.8379 (3)	0.15711 (16)	0.29954 (12)	0.0395 (6)	
H1A	0.8485	0.1042	0.3114	0.059*	
H1B	0.7351	0.1700	0.2890	0.059*	
H1C	0.8855	0.1883	0.3342	0.059*	
N2	1.1282 (3)	0.85669 (15)	0.37681 (12)	0.0384 (6)	
H2A	1.0820	0.8342	0.3372	0.058*	
H2B	1.2330	0.8492	0.3832	0.058*	
H2C	1.1069	0.9104	0.3762	0.058*	
N3	0.8007 (3)	0.88669 (15)	0.42352 (12)	0.0382 (6)	
H3A	0.8401	0.9123	0.4626	0.057*	
H3B	0.6981	0.8759	0.4216	0.057*	
H3C	0.8115	0.9189	0.3886	0.057*	

### Atomic displacement parameters (Å^2^)

**Table:**

	*U*^11^	*U*^22^	*U*^33^	*U*^12^	*U*^13^	*U*^23^
C1	0.0272 (16)	0.084 (3)	0.0303 (15)	−0.0052 (16)	0.0042 (13)	0.0027 (16)
C2	0.0345 (17)	0.0478 (18)	0.0391 (16)	0.0067 (13)	0.0013 (13)	0.0026 (14)
C3	0.0399 (18)	0.0322 (16)	0.0529 (19)	−0.0026 (12)	0.0100 (15)	0.0020 (14)
Cl1	0.0356 (4)	0.0628 (5)	0.0405 (4)	0.0103 (4)	0.0056 (3)	0.0010 (4)
Cl2	0.0291 (4)	0.0586 (5)	0.0487 (4)	−0.0054 (3)	0.0089 (3)	0.0000 (4)
Cl3	0.0263 (3)	0.0409 (4)	0.0391 (4)	0.0016 (3)	0.0038 (3)	−0.0005 (3)
Cl4	0.0340 (5)	0.0407 (6)	0.0420 (6)	0.000	−0.0016 (4)	0.000
Cl5	0.0300 (4)	0.0488 (4)	0.0488 (4)	−0.0043 (3)	0.0055 (3)	−0.0040 (3)
I1	0.03698 (16)	0.03827 (15)	0.02565 (13)	0.000	0.00517 (11)	0.000
I2	0.02793 (11)	0.03197 (11)	0.02788 (10)	0.00081 (7)	0.00317 (7)	−0.00044 (7)
N1	0.0337 (13)	0.0450 (15)	0.0410 (14)	0.0042 (11)	0.0099 (11)	0.0038 (11)
N2	0.0295 (13)	0.0401 (14)	0.0463 (14)	0.0023 (10)	0.0090 (11)	−0.0025 (11)
N3	0.0310 (13)	0.0435 (14)	0.0413 (14)	−0.0039 (11)	0.0103 (11)	−0.0044 (11)

### Geometric parameters (Å, °)

**Table:**

C1—N1	1.463 (4)	Cl2—I2	2.4518 (10)
C1—C1^i^	1.491 (6)	Cl3—I2	2.6790 (9)
C1—H1D	0.9700	I1—Cl1^ii^	2.5595 (10)
C1—H1E	0.9700	N1—H1A	0.8900
C2—N2	1.488 (4)	N1—H1B	0.8900
C2—C3	1.499 (4)	N1—H1C	0.8900
C2—H2D	0.9700	N2—H2A	0.8900
C2—H2E	0.9700	N2—H2B	0.8900
C3—N3	1.483 (4)	N2—H2C	0.8900
C3—H3D	0.9700	N3—H3A	0.8900
C3—H3E	0.9700	N3—H3B	0.8900
Cl1—I1	2.5595 (10)	N3—H3C	0.8900
			
N1—C1—C1^i^	111.4 (3)	Cl2—I2—Cl3	178.30 (3)
N1—C1—H1D	109.3	C1—N1—H1A	109.5
C1^i^—C1—H1D	109.3	C1—N1—H1B	109.5
N1—C1—H1E	109.3	H1A—N1—H1B	109.5
C1^i^—C1—H1E	109.3	C1—N1—H1C	109.5
H1D—C1—H1E	108.0	H1A—N1—H1C	109.5
N2—C2—C3	113.8 (3)	H1B—N1—H1C	109.5
N2—C2—H2D	108.8	C2—N2—H2A	109.5
C3—C2—H2D	108.8	C2—N2—H2B	109.5
N2—C2—H2E	108.8	H2A—N2—H2B	109.5
C3—C2—H2E	108.8	C2—N2—H2C	109.5
H2D—C2—H2E	107.7	H2A—N2—H2C	109.5
N3—C3—C2	114.7 (3)	H2B—N2—H2C	109.5
N3—C3—H3D	108.6	C3—N3—H3A	109.5
C2—C3—H3D	108.6	C3—N3—H3B	109.5
N3—C3—H3E	108.6	H3A—N3—H3B	109.5
C2—C3—H3E	108.6	C3—N3—H3C	109.5
H3D—C3—H3E	107.6	H3A—N3—H3C	109.5
Cl1—I1—Cl1^ii^	179.55 (4)	H3B—N3—H3C	109.5

Symmetry codes: (i) −*x*+2, *y*, −*z*+1/2; (ii) −*x*, *y*, −*z*+1/2.

### Hydrogen-bond geometry (Å, °)

**Table:**

*D*—H···*A*	*D*—H	H···*A*	*D*···*A*	*D*—H···*A*
N1—H1A···Cl1^iii^	0.89	2.65	3.410 (3)	144
N1—H1A···Cl3^iii^	0.89	2.76	3.341 (3)	124
N1—H1B···Cl4	0.89	2.27	3.136 (2)	164
N1—H1C···Cl5^iii^	0.89	2.27	3.148 (3)	168
N2—H2A···Cl4^iv^	0.89	2.38	3.232 (3)	161
N2—H2B···Cl5^v^	0.89	2.26	3.123 (3)	162
N2—H2C···Cl3^iv^	0.89	2.40	3.246 (3)	159
N3—H3A···Cl3^vi^	0.89	2.42	3.297 (2)	167
N3—H3B···Cl5	0.89	2.32	3.144 (3)	154
N3—H3C···Cl1^iv^	0.89	2.49	3.319 (2)	155

Symmetry codes: (iii) *x*+1/2, *y*−1/2, *z*; (iv) *x*+1/2, *y*+1/2, *z*; (v) *x*+1, *y*, *z*; (vi) −*x*+3/2, −*y*+3/2, −*z*+1.
